# A trans-theoretical approach to alcohol abuse profile in the general population of an islamic country - Mashhad, Iran

**Published:** 2015

**Authors:** V Vakili, P Shojaee, A Yaghmaei, Z Abbasi Shaye

**Affiliations:** * Department of Community Medicine, Mashhad University of Medical Sciences, Mashhad, Iran; ** Department of Community Medicine, Legal Medicine Organization, Mashhad University of Medical Sciences, Mashhad, Iran

**Keywords:** alcohol, trans-theoretical model, stages of change

## Abstract

**Background:**From a public health perspective, alcohol-related problems have enormous social and individual consequences.

**Objectives:**The aim of the present study was to apply the TTM on the general population of Mashhad city to evaluate the change levels and possible relative factors regarding alcohol abuse.

**Methods:**: In a cross-sectional design, a total number of 564 people from the General Population of Mashhad, Iran participated. Stages of change questionnaire based on trans-theoretical model (TTM) and the checklist including socio-demographic characteristics as well as possible related factors were used. SPSS 11.5 software was used for all statistical analyses.

**Results: **Among 564 people who took part in this survey, 245 (43.43%) had the history of alcohol consumption or they were current alcohol users. The analysis showed that 19.2% of the participants were in pre-contemplation stage, 3.3% in contemplation, 1.2% in preparation, 2.9% in action, 2% in maintenance and 71.4% were in termination phase. Age, job, smoking, and hookah smoking were identified as predictors of pre-contemplation stage. Marital status, job, and smoking were predictors of termination phase.

**Conclusion: **This picture is from an Islamic holy city where assumed religious beliefs have cramped drinking patterns. According to harm of alcohol abuse, it is necessary to tailor the intervention for target populations. Factors identified as predictors of alcohol abuse such as age, marital status and occupation, as well as being cigarette and hookah smokers should be taken into account in the design of future interventions.

## Background

From a public health perspective, alcohol-related problems have enormous social and individual consequences. Drinking continuum from abstinence, abuse, and dependency to hazardous drinking varied [**1**]. Evidence showed that the health and economic burden of drinking is far greater due to hazardous drinking rather than abuse or dependency [**2**].Generally, alcohol consumption has been reported to be less than 10% in part of the world, but can reach more than 10% in young men in some areas. The prevalence of alcohol dependency is much lower than alcohol use and was estimated to be 0.2% in 2010 [**3**]. 

Religious beliefs have cramped Drinking in Islamic countries such as Iran and Saudi Arabia therefore producing, selling, and drinking alcohol is a punishable crime[**4**] On the other hand, scientific debate and health programming were postponed due to stigma linked to alcohol use in such countries [**5**]

However, alcoholic beverages are available in Iran as a result of black market and illegally homemade products [**6**,**7**] The first national document regarding alcohol misuse was established by the office dedicated to alcohol prevention within the Iranian Ministry of Health and Medical Education in 2013 [**8**].

This study used the trans-theoretical model (TTM) which was developed by Prochaska and DiClemente. The TTM was generalizable across a wide range of problem behaviors as well as a wide diversity of populations with such behaviors. Behavioral change was composed of six stages (pre-completion, completion, preparation, action, maintenance and termination) [**9**-**11**]. The TTM enabled the use of appropriate interventions specifying the stage of change regarding individuals included and increased the success rate [**12**,**13**].

The TTM has been widely accepted and endorsed by researchers and clinicians working in the area of health behavior change [**14**]. Although the model was developed for the use with any health behavior [**15**], little is known to individuals about the applicability of the trans-theoretical model of intentional behavior change (TTM) with unhealthy alcohol consumption [**16**].

## Objectives

The aim of the present study was to apply the TTM on the general population of Mashhad city to evaluate the change levels and possible relative factors regarding alcohol consumption.

## Patients and Methods

This is a cross-sectional study conducted in Mashhad, Iran in 2014. Mashhad is the second largest religious metropolis in the world and the second largest city in Iran. It is the capital of Razavi Khorasan Province and located in the north east of the country, with a population of 2,772,287 at the 2011 population census. A total number of 564 participants participated. The survey was done by using a checklist including socio-demographic characteristics and stages of change questionnaire [**17**]. Stages of change refer to an orderly sequence of changes in alcohol behavior through which people pass according to the TTM. Someone in pre-contemplation (pc) has no intention to quit alcohol consumption within 6 months. A contemplator is someone who still consumes alcohol, but is planning to quit within 6 months. A person in preparation is planning to cease alcohol consumption within 1 month and has taken some initial steps toward it. Someone is called in action when he has quit alcohol consumption for less than 6 months. A person in maintenance has ceased consumption for 6 months or more, and finally the person in termination stage, will never consume alcohol again [**17**]. Therefore, the questionnaire consisted of 6 questions with yes and no responses, according to 6 stages of change.

Demographic information, including age, sex, education level, marital and job status, family size, history of smoking as well as hookah smoking or drug abuse were asked in the checklist. We referred to public transport stations, parking lots, car parks of shopping centers, banks, hospitals, and universities all around the city for data collection. Parking of Imam Reza Holy Shrine was also a place for sampling collection procedure.

The ethics Committee of Mashhad University of Medical Sciences approved the study. The interviewers explained the objectives of research for participants and were assured about the privacy of their personal data and after getting the consent, they filled the checklist.

The SPSS 11.5 software (SPSS Inc., Chicago, Illinois, USA) was used for all statistical analyses. Standard descriptive statistics were applied to describe the pattern of the data. Chi-square test was used to examine the significance of the association between categorical data. The normality of the data was checked with Kolmogorov–Smirnov test. Kruskal-Wallis test was applied in non-normal distributions. The logistic regressions were used to predict factors influencing the pre-contemplation and termination stages. All the tests were 2-tailed, and probability values 0.05 were considered significant.

**Results**

Among 564 people who took part in this survey, 245 (43.43%) had the history of alcohol consumption or they were current alcohol users. 160 consumers were male (65.3%) and 85 were female (34.7%). The mean age of consumers was 31.42 years and the median age was 27. Age of people ranged from 11 to 81. The mean age of women alcohol consumers was 32.46 years and the median was 30, with ranges between 11 and 81. Among men, the mean age was 30.93 years and the median age was 26; and the range was 12-70 years. Details of alcohol consumption according to stages of changes Model mentioned in **Table 1** and **2**. As seen in **Table 2**, most people (71.4%) were in termination stage.

**Table 1:** Frequency of the participant’s stages of change based on trans-theoretical Model (TTM) on alcohol consumption by gender

		Pc (%)	C (%)	P (%)	A (%)	M (%)	T (%)
Age (years)	Mean (med)	29.54 (27)	37.12 (36)	37.33 (42)	28.71 (25)	30.6 (25)	31.68 (27)
Marital status n (%)	single	27 (57.4)	3 (37.5)	2 (66.7)	4 (57.1)	3 (60)	84 (47.7)
	married	13 (27.7)	4 (50)	0 (0)	0 (0)	0 (0)	90 (51.1)
	divorced	7 (14.9)	1 (12.5)	1 (33.3)	3 (42.9)	2 (40)	2 (1.1)
Occupation n (%)	Unemployed and housewife	9 (20.9)	0	0	1 (16.7)	0	42 (25.6)
	employee	1 (2.3)	2 (33.3)	0	1 (16.7)	0	34 (20.7)
	Self employed	22 (51.2)	3 (50)	2 (100)	4 (66.7)	2 (40)	39 (23.8)
	student	11 (25.6)	1 (16.7)	0	0	3 (60)	49 (29.9)
Education n (%)	Less than high school	4 (8.7)	2 (28.6)	1 (33.3)	1 (14.3)	0	16 (9.2)
	High school and more	42 (91.3)	5 (71.4)	2 (66.7)	6 (85.7)	1 (100)	158 (90.8)
Family size	Mean (med)	4.19 (4)	4.12 (4)	2.33 (3)	2.5 (2)	5 (5)	4.14 (4)
Sleep duration (hours a day)	Mean (med)	7.53 (7)	7.37 (8)	6.66 (7)	5.66 (6)	7.8 (7)	7.43 (7)
BMI (kg/ m2)	Mean (med)	23.90 (23.59)	23.73 (23.4)	26.119 (27.17)	22.31 (21.73)	26.97 (26.23)	25.71 (24.16)
Smokers n (%)		35 (74.5)	2 (25)	1 (33.3)	2 (28.6)	2 (40)	23 (13.1)
smoking(pack/year)	Mean(med)	7.08 (5)	6.84 (5)	10 (10)	10 (10)	2.5 (2.5)	7.5 (7.5)
Hookah smokers n (%)		24(51.1)	2 (25)	1(33.3)	0	0	24 (13.6)
Non-addicted n (%)		45 (95.7)	7 (87.5)	3 (100)	7 (100)	5 (100)	175 (99.4)

**Table 2:** Distribution of stages of change on alcohol consumption according to possible related factors among study participants.

	Men N (%)	Women N (%)	Total N (%)
PC	36 (22.5)	11 (12.9)	47 (19.2)
C	5 (3.1)	3 (3.5)	8 (3.3)
P	3 (1.9)	0 (0)	3 (1.2)
A	4 (2.5)	3 (3.5)	7 (2.9)
M	5 (1.9)	0 (0)	5 (2)
T	107 (66.9)	68 (80)	175 (71.4)
*PC: pre-contemplation, C: contemplation, P: preparation, A: action, M: maintenance, T: termination*			

**Table 3:** Predictors of pre-contemplation and termination stage among study participants

		B	S.E.	Wald	df	Sig.	Exp (B)
Pre-contemplation	age	-0.064	0.029	4.827	1	0.028	0.938
	job			7.864	4	0.097	
	unemployed	1.748	0.788	4.921	1	0.027	5.743
	employee	-19.092	5860.693	0.000	1	0.997	0.000
	Self-employed	1.945	0.717	7.349	1	0.007	6.992
	housewife	-17.986	7733.042	0.000	1	0.998	0.000
	nonsmoker	-3.001	0.578	26.906	1	0.000	0.050
	Hookah smoker	-1.160	0.546	4.506	1	0.034	0.314
	Constant	2.171	0.876	6.141	1	0.013	8.767
termination	Marital status			9.808	3	0.020	
	single	-19.365	16414.502	0.000	1	0.999	0.000
	married	-18.640	16414.502	0.000	1	0.999	0.000
	divorced	-21.477	16414.502	0.000	1	0.999	0.000
	job			16.306	4	0.003	
	unemployed	-0.293	0.648	0.204	1	0.651	0.746
	employee	1.802	0.877	4.217	1	0.040	6.059
	Self-employed	-1.275	0.512	6.209	1	0.013	0.279
	housewife	18.801	8235.446	0.000	1	0.998	146249421.128
	nonsmoker	2.323	0.439	28.003	1	0.000	10.210
	Constant	18.922	16414.502	0.000	1	0.999	165151856.793
Cox & Snell R Square1: 0.356, 0.348							

Gender was not statically different on various stages of change (p=0.13). There was a significant statistical relationship between the marital status and alcohol TTM stages (p=0.00) and married people were more at termination. Among singles, there were 5 widows all being at termination stage. However, there was no statistically significant relationship between alcohol TTM stages and education (p=0.33). The numbers of more educated people were more at any stage compared to less educated people. The only illiterate person in this survey who was a 70-year-old man was at termination stage. There was a statistically significant relationship between Hookah smoking and alcohol TTM stages (P=0.00). Hookah smoking was more common at pre-contemplation stage. Cigarette smoking and alcohol consumption (regardless of alcohol TTM stage) have a statistically significant relationship (p=0.00). There was no statistically significant relationship between alcohol TTM stages and age (p=0.74), family size (p=0.08), sleeping hours per day (p=0.34), smoking amount (pack/ year) (p=0.15B) and BMI as well (P=0.39). There was a statistically significant relationship between job and stages of change on alcohol (p=0.007). Employees and students were more likely tempted to quit alcohol. 24 participants were housewives and all were at termination. Age, cigarette smoking and hookah smoking, were identified as predictors of pre contemplation stage. Being unemployed and self-employed were predictors of pre-contemplation stage. Unemployed people were 5.7 times more than students in pre-contemplation stage (P=0.02) and self-employed were 6.99 times more than students in pre-contemplation stage (p=0.00). The marital status, job, and smoking were predictors for termination stage. Most employees with governmental jobs were 6 times at alcohol termination stage compared to students (p=0.04). Nonsmokers were 10.2 times more likely to terminate alcohol compared to smokers(**Table 3**).

We asked people if they had an Encourager to quit alcohol, and there was a significant statistical relationship between having a motivator to quit and alcohol TTM stages (P=0.02). However, there was no statistical relationship regarding the person who encouraged people to quit (a friend or family member or consultant) (p=0.857). Therefore, it is not important who motivates people to quit alcohol and abstention. People, who were aware of alcohol disadvantages, were more likely to quit alcohol (P=0.00). There was a statistically significant relationship between family history of alcohol consumption and alcohol TTM stages (P=0.00). People without a family history of alcohol consumption were predominantly more likely at termination stage. There was a significant statistical relationship between the onset of alcohol abuse at school age and alcohol TTM stages (P=0.00). People began alcohol consumption at school ages and were not eager to simply quit alcohol. 64.1 of the pre-contemplators had predicted their problematic alcohol abuse at the onset of drinking and finally the awareness of the existence of consultant institutes had a statistically significant relationship with alcohol TTM stages (P=0.04).

## Discussion

In this survey, 43.43% of the participants reported previous or present history of alcohol consumption. However, most people with history of alcohol consumption had quit and were in the termination stage according to TTM stages of change model. It can be due to Islamic religious beliefs in Iran and social condition in which alcohol consumption is not acceptable [**25**, **28**]. 

Women were more likely to quit alcohol (66.9% in men vs. 80% in women at termination stage) and men were more likely to continue consumption (22.5% in men vs. 12.9 in women at pre-contemplation stage), most surveys done all over the world showed that drinking is more prevalent among men than among women and abstention is more prevalent among women [**18**-**21**, **30**-**38**]. Even in communities where female and male alcohol abuse prevalence was the same, males had worse alcohol abuse habits specially risky and heavy drinking [**22**-**25**,**29**,**36**, **38**,**39**]. The proportion of alcohol consumption between men and women varied according to the region, culture of people, religion, etc., but men were exceeding than women in most studies, and, according to some literature data regarding the level of alcohol abuse they were even three times more [**27**,**29**,**30**] and women were more abstainers [**40**].

The marital status was a robust predictor of alcohol consumption in young adulthood [**41**]. Marriage decreases alcohol consumption [**30**,**31**,**35**,**41**-**44**]. In this survey, single people were more likely to use alcohol (57.4% of singles vs. 27.7% of married who were in pre-contemplation stage), but there was not such a big difference between singles and married people at termination stage (44.9% of singles vs. 51.1 of married at termination stage).Although these differences were all significant and the marital status was an important factor for alcohol abuse (p=0.00), the widows in our study were all woman and at termination stage. There were different results in different surveys about widows’ alcohol consumption,some were the same as our result [**32**,**35**,**45**] and the other was against [**46**]. There was not enough number of cases to find the relationship between getting divorced and alcohol consumption in our survey and further studies are needed to investigate this relationship.

Family size was not different between alcohol TTM stages. Some surveys showed having children was an encouraging factor to quit alcohol [**39**].

Occupation was an important factor for alcohol TTM stages. Unemployed people were 5.7 times more than students at pre-contemplation stage. Self-employed people were 6.99 times more than students at pre-contemplation stage. People with governmental jobs had 6 times more at alcohol termination compared to students. Employees and students were more likely at termination stage, while people who were on their own jobs without governmental employment were at pre-contemplation stage. Many studies showed a relationship between job and alcohol consumption pattern but most focused on job according to income and the results in most studies revealed that high-income jobs were associated with more alcohol consumptions [**26**,**35**,**37**,**40**,**47**,**48**]. All housewives quit alcohol in our survey, and thiswas reported in some surveys as well; and the reason could be due to the nature of the housework that was fairer than other jobs with lower conflict levels and the roll of satisfactory marital communication with partner as well as commitment to familial responsibilities [**49**]. According to some papers being unemployed contributed to alcohol consumption [**29**], but others were against this and showed that alcohol consumption was more among the employed ones [**37**] although in these studies authors suggesteda relationship between consumption and economic status and income [**26**,**37**,**40**,**47**,**50**,**51**]. Also, lower educated people tended to worsen the consumption habits such as heavy drinking [**47**,**51**]. In our survey, unemployed tended to use alcohol more and the same as Fone DL et al. mentioned in 2013, unemployed people were more alcohol users [**36**]. In our survey, there was no relationship between education and alcohol consumption, but findings showeda total alcohol consumption, regardless of alcohol TTM stages, being more prevalent among well educated people. Keeping in mind the low number of participants, however such a result has been mentioned in several surveys, in which higher-educated people tended to use alcohol more than lower-educated ones [**35**,**39**,**40**,**50**,**51**]. Although according to most surveys higher education was related to more alcohol consumption in average, but lower educated people had worse alcohol consumption habits such as heavy drinking and they showed worse results such as more fatalities due to alcohol overuse [**51**,**52**]. Relationships between addiction and alcohol TTM stages was not statistically significant and needed further investigation; more participants and hookah smoking were identified as predictors of pre contemplation stage. Near a quarter of people at pre-contemplation stage were smokers while a small amount of people at termination stage, were smokers. Therefore, smoking was significantly more common among people who drank alcohol. Several surveys showed the relationship between drinking and smoking. Hookah smoking was statistically important and people in termination phase were more likely not hookah smokers [**38**,**44**,**48**], but the amount of smoking (pack /year) was not statistically significant in this study. Further studies warranted attention. Sleep hours per day and BMI were not related to alcohol TTM stages in this study. People, who were aware of alcohol disadvantages, were more likely to quit alcohol, most of the people at termination stage were aware of the disadvantages. Therefore, it seemed that adequate information to population was an effective method. Family history of alcohol abuse was a strong predictor and most of the people at termination were without a family member using alcohol. Previous studies indicated a family history of alcohol addiction as a strong predictor of alcoholism as well [**49**].

While having an encourager to quit was a predictor of the termination stage, it was not important who encouraged them to quit. Anyone could be helpful. This may be due to the low number of participants in these categories in the present study. Further investigations with more sample sizes were appreciated to the better understanding.

People who began alcohol consumption at school ages did not tend to quit alcohol. Underage drinking was thought to be a powerful predictor of later adult alcohol abuse and alcohol dependence in previous studies as well. Near half of the pre-contemplators had predicted their problematic alcohol abuse at the onset of drinking.

The observed relation among alcohol abuse stages of changes and the determinants should interpret with caution and because of cross-sectional design that all variables were measured simultaneously, their association not necessarily establishing the causation. On the other hand, data were subjective and self-reported. It might result in over or under reporting. Longitudinal designs with objective measures would be of interest in future studies. However, this was the first study to investigate the Stages of change alcohol abuse and related factors in Iranian adults. On the second thought, data collection by trained interviewers and unawareness of them from the purpose of the study strengthened the results. Representativeness was assured through random selections of public areas across the city.

Near half of the participants reported previous or present history of alcohol consumption and only half were lifelong abstainers. This picture is from an Islamic holy city where assumed religious beliefs have cramped drinking patterns.According to the harm of alcohol abuse, it was necessary to tailor intervention for target populations. Factors identified as predictors of alcohol abuse such as age, marital status and occupation as well as being cigarette and hookah smokers should be taken into account in the design of future interventions.

**Acknowledgements**

We kindly appreciate the efforts of all the people involved in the project for the recruitment of participants and collection of the data. This project is sponsored by Mashhad University of Medical Sciences.

**Funding/ Support **

This study was supported by Mashhad University of Medical Sciences, Mashhad, Iran.
